# Magda Nico and Gary Pollock (Eds), *The Routledge Handbook of Contemporary Inequalities and the Life Course*

**DOI:** 10.1007/s10680-024-09700-y

**Published:** 2024-05-28

**Authors:** Anne McMunn, Joseph Harrison

**Affiliations:** 1 Research Department of Epidemiology & Public Health, University College London, London, WC1E 7HB UK; 2 School of Geography & Sustainable Development, University of St Andrews, St. Andrews, KY16 9AL UK

*The Routledge Handbook of Contemporary Inequalities and the Life Course* is a highly ambitious edited collection which uses a vast range of disciplinary and methodological perspectives.  By using these perspectives, it builds on previous life course inequality research. The editors, Magda Nico and Gary Pollock, make clear the aim of the volume is to challenge the notion of inequalities being static. Life course research, with its focus on trajectories and transitions across interrelated domains, is a natural framework for advancing their aim. This is a timely contribution to an important topic. For example, income inequality in the UK is greater now than it was 50 years ago and remains persistent (Francis-Devine & Orme, 2023). Taking a life course approach enables the identification of traction points and critical periods in which trajectories of disadvantage can be altered.

This volume is comprised of 32 chapters organised into eight sections giving tremendous breadth and depth to the topic, presenting an array of dimensions of inequality and life course domains. Early sections offer theoretical considerations for conceptualising, measuring, and interpreting life course inequalities, and the analytical methods that can be used. Later sections cover specific types of inequalities such as health, racial, economic, and gender. The temporal aspects of life course inequalities also have close attention paid to them. Readers will recognise the names of many familiar leaders in life course research as well as contributions from scholars working in complementary areas of inequalities research that may be unfamiliar to a life course readership. This multidisciplinary approach, bringing together scholars from sociology, psychology, economics, public health, statistics, and more, is one of the key contributions of the volume, allowing for a nuanced and complex understanding of how inequalities operate across different domains of life, building from theoretical and methodological to more applied perspectives.

A key strength of the collection is its pervading recognition, and demonstration, of the dynamic complexity of life course inequalities. This is demonstrated in the first section which covers inequalities as process. The editors preface each section here and provide a compelling description of inequality as interlocking trajectories of life domains and institutions travelling from, through, and to particular identities and positions. While the study of life course inequalities is not new, it becomes clear from the outset that this collection will provide a wider, highly dynamic framing of the topic. The four chapters of this first section provide an excellent introduction to key life course concepts and processes. Chapter 1 introduces the key concept of cumulative (dis)advantage (usually abbreviated to CAD, here CDA) over the life course within the context of temporal increases in inequality which are currently a concern for many. Addressing poverty from a life course perspective, Chapter 2 shows the importance of duration of exposure and the ‘spillover’ of poverty-related stress into other life domains—a cornerstone of health inequalities research. Chapter 3 notes that income is an inadequate measure of inequality as a process. The chapter draws on Sen’s capability approach to ask whether the intergenerational transfer of inequality means inequality of opportunity—rather than outcome—is where resources should be targeted to reduce inequalities. Focusing on policy environments, Chapter 4 demonstrates that UK cohort data has helped to provide evidence on ‘what works’ in relation to child well-being and inequalities. Notably, the chapter equates notions of meritocracy with victim blaming and comments on concerns expressed by some that welfare state provision disincentivises work.

Section 2 argues that multiple, complementary methods are required for assessing life course inequalities, although all the chapters in this section are quantitative in focus. Chapter 5 sets out Elder’s five principles of life course research and explains the importance of longitudinal life course data for studying social mobility, which will come as no surprise to life course researchers. Using sequence analysis (a popular tool in life course research) on the Swiss Household Panel, Chapter 6 describes gender differences in family trajectories and then uses Markov models to investigate life course transitions that may predict work and family group membership. As shown in many other countries, they find that work-family life courses remain traditional and gendered in Switzerland. One interesting aspect of the book is that it was written as the COVID-19 pandemic arose and was completed while it was still in full-swing. Chapters 7 and 8 are the first in the book to focus on this topic. Chapter 7 uses multi-level models to look longitudinally at area inequalities in COVID-19-related outcomes in Brazil. Chapter 8 provides an introduction to some of the key statistical challenges in dealing with types of confounding and bias, acknowledging that COVID-19 can exacerbate these too.

The third section concentrates on social stratification and health which is a vast area of research across the social sciences as well as public health and social epidemiology, including the entire sub-discipline of life course epidemiology. Rather than revisiting these well-trodden areas, after Chapter 9 which is a classical review of socioeconomic inequalities in mental health from a life course perspective, the remaining three chapters bring novel applications. Chapter 10 suggests that a focus on average life expectancy masks variations in the distribution of age at mortality that can be captured with the concept of ‘lifespan’ inequality. The chapter demonstrates cohort differences in the age distribution of mortality to show the importance of this concept. The neglected topic of disability and partnerships is highlighted by Chapter 11, using Swedish register data to show that people with disabilities continue to be excluded from partnership formation. This has well-known associations with health and well-being and the fact that very little improvement is found even in the relatively equitable context of Sweden is jarring. The final chapter of this section is a reprint of an overview of life course inequalities in the context of the pandemic which was written by an all-star cast of well-known authors in this field. It was written at the height of the pandemic and, while it is interesting to reflect on what we have learned since it was written, their assertion that the “pandemic has also served as a reminder that health and well-being are not only individual characteristics but public goods that matter for the welfare and functioning of whole communities” (p. 167) remains true today.

In some of the chapters of Sects. 4 and 5, life course material is less obvious. Section 4 is on economic and wealth inequalities. Chapter 13 begins as a primer on the basic concepts and measures of social stratification and develops into a fascinating in-depth discussion of both theoretical and empirical aspects of social reproduction. Chapter 13 finishes on ‘horizontal’ inequalities which lead nicely to the topic of measuring intersectional inequalities in life course research covered in Chapter 14. One useful exercise in reading the collection is recognising differences in the ways authors deal with the same concept such as social stratification. Here, different dimensions of socioeconomic position are described with perhaps less recognition of theoretical and conceptual differences between them. The final two chapters of this section do not feel entirely at home in a collection on life course inequality. Chapter 15 is a macro-economic treatise on Piketty’s views on capital while Chapter 16 argues that inequalities rise naturally from a capitalist system; the concept of the life course appears as a bit of an after-thought here. The fifth section covers particular stages of the life course: youth, education, and transitions to adulthood. Both chapters 17 and 18 use an empirical cross-sectional analysis of time trends to investigate education expansion. Although the first focuses on Germany while the second is internationally comparative, perhaps these two chapters could have been combined. Chapter 19 is an intriguing history of occupational class and working lives in post-war Britain and Chapter 20 is a qualitative picture of classed youth identity in Norway. Many of the chapters in this collection tackle meaty theoretical aspects of social class in a clear and accessible manner and Chapters 19 and 20 are among the best in this regard.

Section 6 focuses on family and ‘linked lives’, one of the central concepts of life course research, which refers to the fact that each life course is fatefully influenced by the decisions and events in the life courses of those around them (Elder, 1994). The introduction to the section successfully describes the dense web of similarities and differences at play within families and notes that “social stratification *is* a family issue” (p. 272). Chapter 21 is an empirical investigation of unpaid family care in the Survey of Health, Retirement and Ageing in Europe (SHARE). Confusingly, the terms care, social support and inclusion/exclusion are used interchangeably in this chapter. Using a measure of ‘balance’ between the amount of care participants give and receive, the authors investigate predictors of being in the bottom quintile of giving more than one receives, although it is not mentioned that being in receipt of care is normally driven by limitations to function brought on by an illness or condition. In its qualitative investigation of the family as the unit of analysis, Chapter 22 makes powerful theoretical contributions on the complexities of capitals exchanged within families and the role of the family in mediating and moderating the individual’s experience of their social strata, observing that “It is the stubbornness of time, unidirectional, and the accumulating episodes and events of life that overcome the inertia of structure and produce social change.” (p. 285). The authors argue that the family should become a fixed level of analysis between the individual and the supra-individual in Bernardi and colleagues’ life course ‘cube’ (Bernardi et al., 2019). Chapter 23 is an example of classic life course research; it uses fixed-effects models to investigate the association between family formation and occupational class, and whether this differs by class of origin, in Italian survey data. The chapter finds, as has been shown elsewhere, that female careers are penalised by entry to parenthood, driven primarily by career interruptions. The final chapter in this section (Chapter 24) is an entirely conceptual piece on late (age 40 +) fertility in relation to normative timing pressures and narratives of risk.

Gender inequality is the topic of Sect. 7, explored in the binary, heteronormative sense, as well as a time-varying construct. This section stands out for its novelty in introducing elements of queer temporalities to combat heteronormative approaches to life course research. Chapter 25 is an engaging discussion of gender and sexuality as social structures, which highlights both the need to build bridges between these two research communities as well as challenge the assumptions that those within the LGBTQ + umbrella share the same struggles as each other. Warnings here on treating intersectionality as a mere addition to pre-existing social categories are informative for attempts to study intersectionality quantitatively and lead nicely to Chapter 26, which takes gender change seriously as a unit of measurement in quantitative life course research. This novel approach reflects gender hierarchies that are complex and nonlinear. It uses empirical qualitative methods to document the gender trajectories of transitioned people in the UK, Sweden, and Portugal, including the experiences of trans men recognising new-found male privilege alongside acceptance by self and others. This chapter is an exceptionally interesting, novel addition to life course research. Chapter 27 covers the topic of work—traditionally a highly gendered life course domain—and the intersectional life course, touching on the role of socialisation and micro-processes in producing structural inequality. The final chapter in this section works in broad brushstrokes, drawing on the work of Alwin (2012) to essentially pit sociologists, who “try to grasp the flexibility of human life” (p. 362), against the supposed essentialist and deterministic approaches of biology, psychology, and human development disciplines in the context of LBGTQ + life course temporalities.

The eighth and final section focuses on racial and ethnic inequalities and is an excellent example of the diversity of voices included in this collection. Chapter 29 introduces life course researchers to the discipline of world-systems research studying the role of periphery, semi-periphery, and core areas in the colonial world system where “Whiteness is just as much a geopolitical category as it is a racial designation” (p. 380). Using UK Census data, Chapter 30 illustrates the role of three life course concepts—historical time, CAD, and linked lives—in relation to ethnic health inequality in later life and concludes that there has been little scholarship on the experience of ageing in ethnic minority groups partly due to a lack of representation in quantitative longitudinal data sets. As with Chapter 29, Chapter 31 focuses on variations in historical time and space rather than the life course, using a colonial framing to inform our understanding of inequality. The authors encourage disciplines outside economics to provide diversity and nuance to understandings of inequality. The final chapter of the book returns to the topic of the COVID-19 pandemic exacerbating existing vulnerabilities of marginalised groups (here asylum-seekers, refugees, and migrants)—vulnerabilities which are generated by the social determinants of health according to the socio-behavioural lens of this chapter.

The collection, as a whole, is successful in combining novel approaches with material from more traditional life course perspectives. The focus often leans more towards inequalities than the life course; however, even the chapters with more tenuous links to the life course expose readers to new, unexpected vantage points which enrich the reading experience. Exposure to such diversity is likely to benefit the field of life course research. The book also offers examples of various methodologies that can be used to capture life course inequalities, such as sequence analysis, multi-level modelling, and longitudinal qualitative work, and is highly topical with its considerations of COVID-19 as an exogenous factor that both heightened pre-existing inequalities as well as producing new ones. Each chapter is purposeful and illustrative of the topic it covers, and, generally, the main contribution or argument of each chapter is clear.

While its heterogeneity is in many ways this collection’s advantage, the handbook does lack a common thread through chapters which makes it difficult to consider and interpret the work as one whole piece. While it is virtually impossible for such a diverse range of theoretical, empirical, multidisciplinary, and multi-methods contributions to entirely cohere, it means that every reader, regardless of experience or background, will learn something new.
